# Neural Basis of Number Sense in Larval Zebrafish

**DOI:** 10.1101/2024.08.30.610552

**Published:** 2024-09-05

**Authors:** Peter Luu, Anna Nadtochiy, Mirko Zanon, Noah Moreno, Andrea Messina, Maria Elena Miletto Petrazzini, Jose Vicente Torres Perez, Kevin Keomanee-Dizon, Matthew Jones, Caroline H. Brennan, Giorgio Vallortigara, Scott E. Fraser, Thai V. Truong

**Affiliations:** 1 Translational Imaging Center, Michelson Center for Convergent Bioscience, University of Southern California, Los Angeles, CA, USA; 2 Molecular and Computational Biology, University of Southern California, Los Angeles, CA, USA; 3 Quantitative and Computational Biology, University of Southern California, Los Angeles, CA, USA; 4 Centre for Mind/Brain Sciences, University of Trento, Rovereto, Italy; 5 School of Biological and Behavioral Sciences, Queen Mary University of London, London, United Kingdom; 6 Joseph Henry Laboratories of Physics, Princeton University, Princeton, NJ, USA

## Abstract

Number sense, the ability to discriminate the quantity of objects, is crucial for survival. To understand how neurons work together and develop to mediate number sense, we used two-photon fluorescence light sheet microscopy to capture the activity of individual neurons throughout the brain of larval *Danio rerio*, while displaying a visual number stimulus to the animal. We identified number-selective neurons as early as 3 days post-fertilization and found a proportional increase of neurons tuned to larger quantities after 3 days. We used machine learning to predict the stimulus from the neuronal activity and observed that the prediction accuracy improves with age. We further tested ethanol’s effect on number sense and found a decrease in number-selective neurons in the forebrain, suggesting cognitive impairment. These findings are a significant step towards understanding neural circuits devoted to discrete magnitudes and our methodology to track single-neuron activity across the whole brain is broadly applicable to other fields in neuroscience.

## Introduction

Understanding quantity, whether discrete (countable) or continuous, is fundamental for survival, e.g. for avoiding predators, finding food, mating, and other group behaviors (Alder & Rose, 2000; Cross & Jackson, 2017; Edwards et al., 2002; Templeton & Greene, 2007). Quantity estimation, often referred to as the Approximate Number System (ANS), allows both humans and animals to intuitively estimate numerosity, or quantity of objects in a set, without precise counting (Brannon & Merritt, 2011; Piazza, 2010; Piazza et al., 2004). The ANS develops during early infancy, highlighting its importance as a foundational aspect of cognition (Xu & Spelke, 2000). The ANS forms the basis of survival instincts to complex mathematical abilities, ultimately shaping how we perceive and interact with the world (Bonny & Lourenco, 2013; Feigenson et al., 2004; Szkudlarek & Brannon, 2017).

Current studies are limited to identifying individual neurons or specific brain regions responsible for number sense, but understanding how a network of number-selective neurons functions across the entire brain remains elusive (Ditz & Nieder, 2015, 2016; Messina, et al., 2022a, 2022b; Pfeifer et al., 2018; Piazza et al., 2004; Viswanathan & Nieder, 2013). In primates, Viswanathan & Nieder (2013) showed a visual sense of number mapped to the parietal and prefrontal cortices. Expanding on this, recent studies suggest that visual number processing extends beyond these regions and involves the superior colliculus, a deep subcortical area (Collins et al., 2017; Georgy et al., 2016). In birds, involvement of several pallial regions has been recently documented by early gene expression (Lorenzi et al., 2024). While the use of electrodes can access individual neurons at multiple regions, it is difficult to unbiasedly capture all neurons especially without neuron damage after implantation (Eles et al., 2018; Ferguson et al., 2019; Goss-Varley et al., 2017). Capturing neuronal activity across the whole brain would enable researchers to map neural circuits involved in ANS processing with unparalleled precision in both encoding and representation..

To address this, we developed and optimized a two-photon fluorescence light sheet microscopy (2P-LSFM) platform (Keomanee-Dizon et al., 2020; Messina, Potrich, Perrino, et al., 2022; Truong et al., 2011; Vito et al., 2020) and a customized data analysis pipeline. This allowed for noninvasive imaging of the functional activity of nearly all neurons across the whole brain in larval zebrafish (*Danio rerio*) with single-neuron resolution. Larval zebrafish offer many advantages as a model system for studying neural processes such as transparency, genetic tractability, and drug screening applications (Kawakami, 2005; Lubin et al., 2021; Trinh & Fraser, 2013; Weber & Köster, 2013). By expressing a nuclear-localized calcium indicator (H2B-jGCaMP7f) (Dana et al., 2019a), we were able to monitor whole-brain neuronal activity in response to non-symbolic, visual numerical stimuli. While no studies have shown any ability to discriminate different numerosities at an early age (<7 days post-fertilization (dpf)) in zebrafish, it must develop before the behavior becomes apparent (Adam et al., 2024; Lorenzi et al., 2023; Lucon-Xiccato et al., 2023; Sheardown et al., 2022a). Here, we aim to identify these number-neurons, across the brain, in real time as the animal processes a visual number stimulus, to improve our understanding of the neural basis of number sense.

## Result

**Figure 1.**
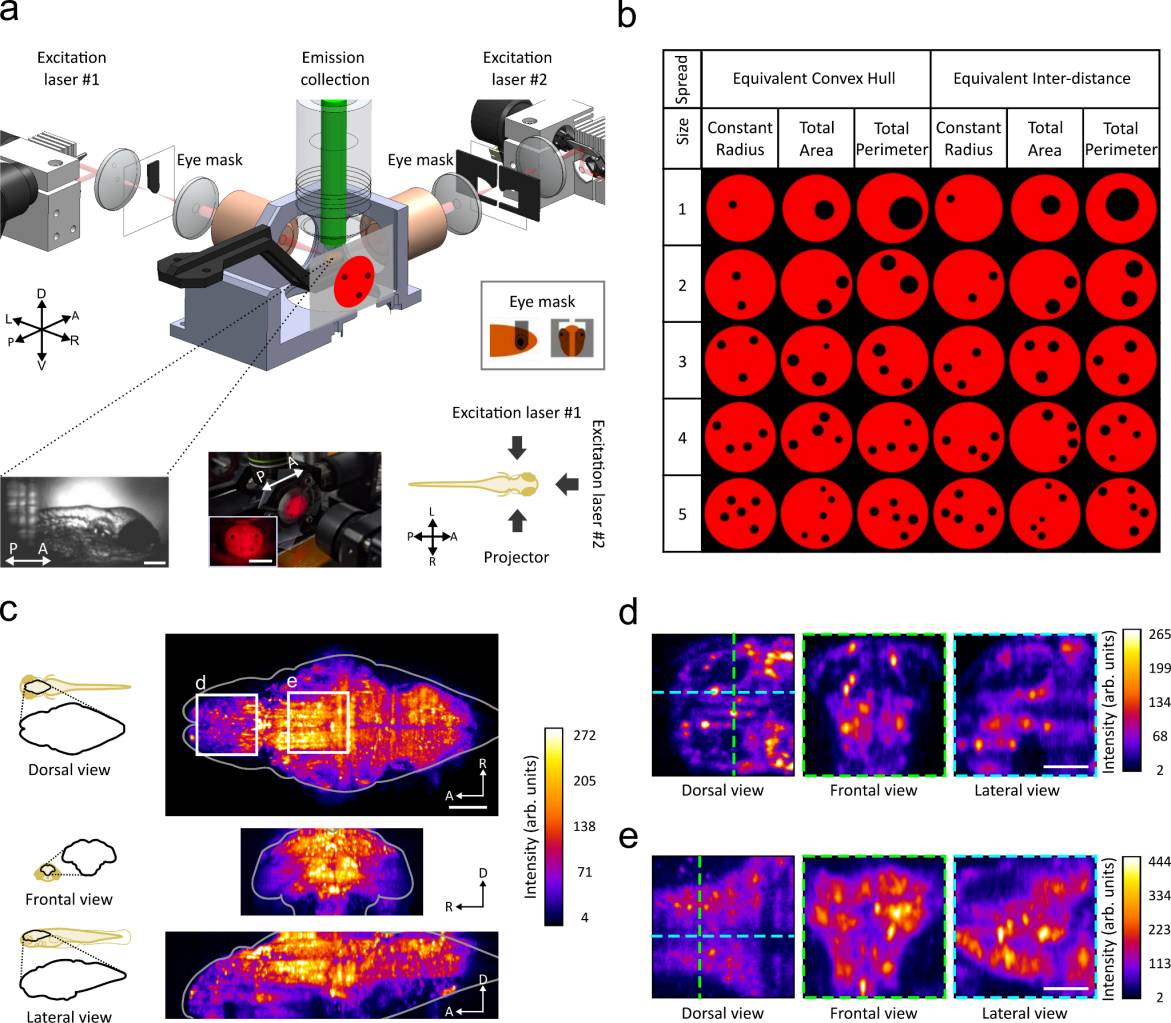
Application of two-photon fluorescence light sheet microscopy to detect neuronal representation of number perception in larval zebrafish. **a** Schematic of the two-photon light sheet fluorescence microscopy system. The sample is excited by two orthogonal 920 nm scanning lasers to obtain uniform excitation of the labeled neurons (Methods: Calcium imaging). A physical eye-shaped mask blocks the laser illumination of the eye. Fluorescence is detected by an objective above the sample. The visual-based number stimulus is displayed on the right side of the larval fish. Bottom-left: brightfield image of the mounted sample, scale bar = 200 μm. Center-bottom: stimulus projection image, scale bar = 10 mm. Bottom-right: Dorsal view of the sample relative to the stimulus direction and excitation laser. A = anterior, P = posterior, D = dorsal, V = ventral, L = left, R = right. **b** Examples of different continuous geometric parameters used to control for co-varying non-numerical variables when changing quantities of objects. Convex hull and inter-distance controls spread of the dots, while radius, total area and perimeter controls for the dot size. See Method: Stimuli Generation. **c** Example fluorescence maximum image projection (MIP) of a 7 dpf zebrafish brain. Top, dorsal view, maximum projection along the dorsal/ventral axis, white boxes area depicted in (d) and (e); middle, frontal view, MIP along the rostral/caudal axis; bottom, lateral view, MIP along the left/right axis. MIPs are averaged over 60 seconds. Scale bar = 100 μm. **d,e** Example magnified fluorescence image showing cellular resolution. (d) forebrain (e) tectum. Left, dorsal view of a single plane; middle, frontal view of a single plane along the green dash lines; right, lateral view of a single plane along cyan dash line. Scale bar = 50 μm.

We used our custom-developed 2P-LSFM to record whole-brain neuronal activity of agarose-embedded zebrafish at age 3, 5, and 7 dpf, while the animals were presented with visual numerical stimuli based on dots (Figure 1a, Methods: Calcium imaging). Imaging was acquired at a 1-Hz whole-brain volumetric rate, and the entire imaging experiment lasted for 90 minutes, as the numerical stimuli sequenced from one to five dots. When the quantity of dots changes, non-numerical geometric effects co-vary and can confound the numerical effects. For example, two circles have a higher combined area than one circle of the same diameter. To account for geometric effects, the number-based dot stimuli controlled for both numerical and non-numerical variables (i.e. for continuous physical variables that co-vary with numerosity) (Zanon et al., 2022). The non-numerical variables were divided into spread and size of the individual dots. The Angular diameter of the dots was kept at a minimum of 5° (angular degree) above the visual acuity threshold of 2–3° (Haug et al., 2010). The spread of the dots includes convex hull and distance between the dots, while the dot size includes constant radius, total dot area, and total dot perimeter (Figure 1b). The stimuli sequence includes all possible combinations of spread and sizes using a new pattern for each stimulus (Supplementary Figure 1). To account for any intrinsic neural oscillatory signal that might have a repetitive pattern in phase with the stimulus display, the inter-stimulus interval followed a pseudo-random sequence.

We applied and optimized several publicly available Python tools and software for managing, processing, and analyzing volumetric movie data and neuronal signals. We used VoDEx (Nadtochiy et al., 2023) to manage the 4D (volumetric movie) data and stimuli annotations. Advanced Normalization Tools (Avants et al., 2009) was used to spatially align and correct for motion artifacts in 4D datasets. Registration of multiple samples onto a representative brain template was performed using ITK-SNAP (Yushkevich et al., 2006).

To segment for the signals from individual neurons, we applied the Python toolbox for large-scale Calcium Imaging Analysis (CaImAn) (Giovannucci et al., 2019). See Methods for full details. Figure 1c, d, e shows example images of the raw image data with whole-brain coverage at cellular resolution. The resulting processed segmentation is shown in Supplementary Figure 2.

### Neurons correlating to number stimuli are detected early in development

**Figure 2.**
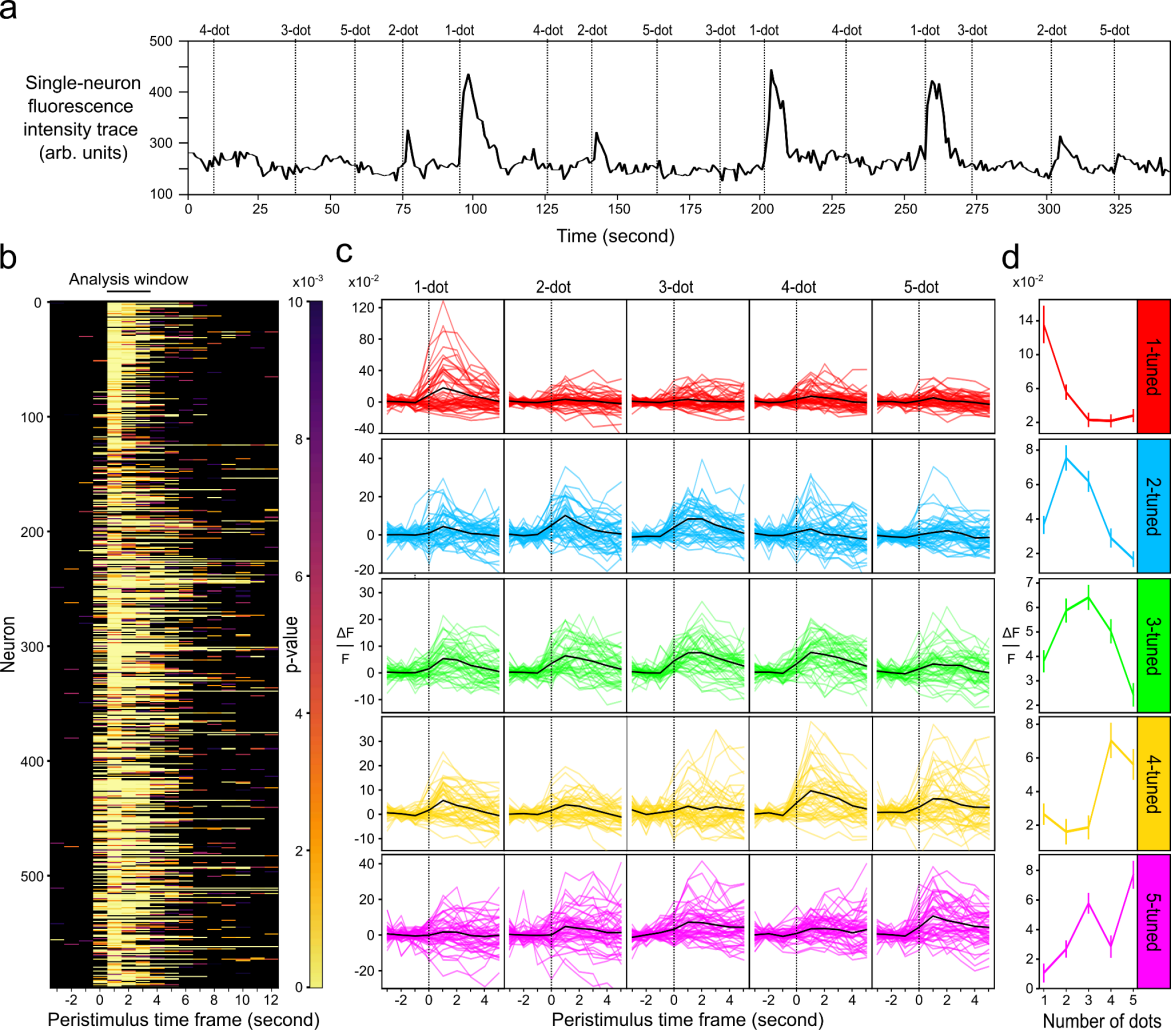
Number-selective neurons produce higher Ca^2+^ activity for preferred numerosities. **a** Example raw fluorescence intensity trace of Ca^2+^ activity from a single neuron during numerical stimuli. Neurons responsive to numerical stimuli show a Ca^2+^ spike after the stimulus onset. **b** Statistical significance of number selectivity over time during stimulus onset in one example 7 dpf larvae. Rows represent individual neurons (n = 599). Significance of number selectivity is centered around the stimulus onset (peristimulus). Stimulus starts at time = 0 and lasts for 1 second. Colormap indicates the p value of selectivity to the stimulus. Black solid line on top indicates a 3-second analysis window used to detect number-selective neurons with a two-way permutation ANOVA to account for the rise and decay time of the Ca^2+^ response. **c** Ca^2+^ activity trace of 5 example neurons with preference to 1–5 dots. Traces are centered around the stimulus onset (peristimulus) for preferred and non-preferred numerosities. Neuron preferences were selected by the highest average activity for a numerosity (for example traces to nonnumeric geometric effects see Supplementary Figure 3). Top labels indicate the number of dots presented, and each color represents the specific number tuning (1 = red, 2 = cyan, 3 = green, 4 = yellow, 5 = magenta). Baseline for *Δ*F/F is calculated by averaging the 3 time points prior to the stimulus onset (dotted vertical line). Black line indicates the average across 48 trials. **d** Tuning curves of each of the five neurons from **c.** Each entry is the average of the 3-second analysis window for each numerosity presentation (n = 3 * 48 numerosities). Error bar = SEM.

To identify neurons specifically responsive to changes in numerosity (Figure 2a, Supplementary Figure 3) from those responsive to geometric changes, we applied a two-way permutation ANOVA. Neurons were filtered based on a significant main effect for changes in numerosity (p < 0.01), without exhibiting significant main or interaction effects due to geometric changes. We define these filtered neurons as “number-selective neurons”. As an example, we present the significant main effect for changes in numerosity during the stimulus onset for a 7 dpf larva (Figure 2b). The analysis window of 3 seconds accounts for the typical ~2 second decay time constant (Dana et al., 2019b; Yang et al., 2022). In general, a significant Ca^2+^ response to a numerical stimulus was detected 0–3 seconds from the stimulus onset.

On average, we identified 1300±300, 800±100, 550±100 number-selective neurons, among 14000±2700, 17000±1300, 17000±2000 detected active neurons in 3, 5, and 7 dpf larval zebrafish, respectively (n=5 for each age group) (Supplementary Table 1–5). Due to the varying expression levels of H2B::GCaMP across individual fishes and varying signal-to-noise ratios due to development of the skin (Li & Uitto, 2014) and head (Kimmel et al., 1995), quantification of number-selective neurons are normalized by number preference, detected active neurons, and regions for each sample. Example Ca^2+^ signal traces and tuning curves of neurons tuned to 1–5 objects for one fish are shown in Figure 2c. Neurons showing a peak Ca^2+^ response to a specific numerosity is defined as tuned or having preference to that numerosity. Tuning curves show a gradual decrease in Ca^2+^ response for numerosities further from the preferred numerosity (Figure 2d; for population tuning see Supplementary Figure 4).

**Figure 3.**
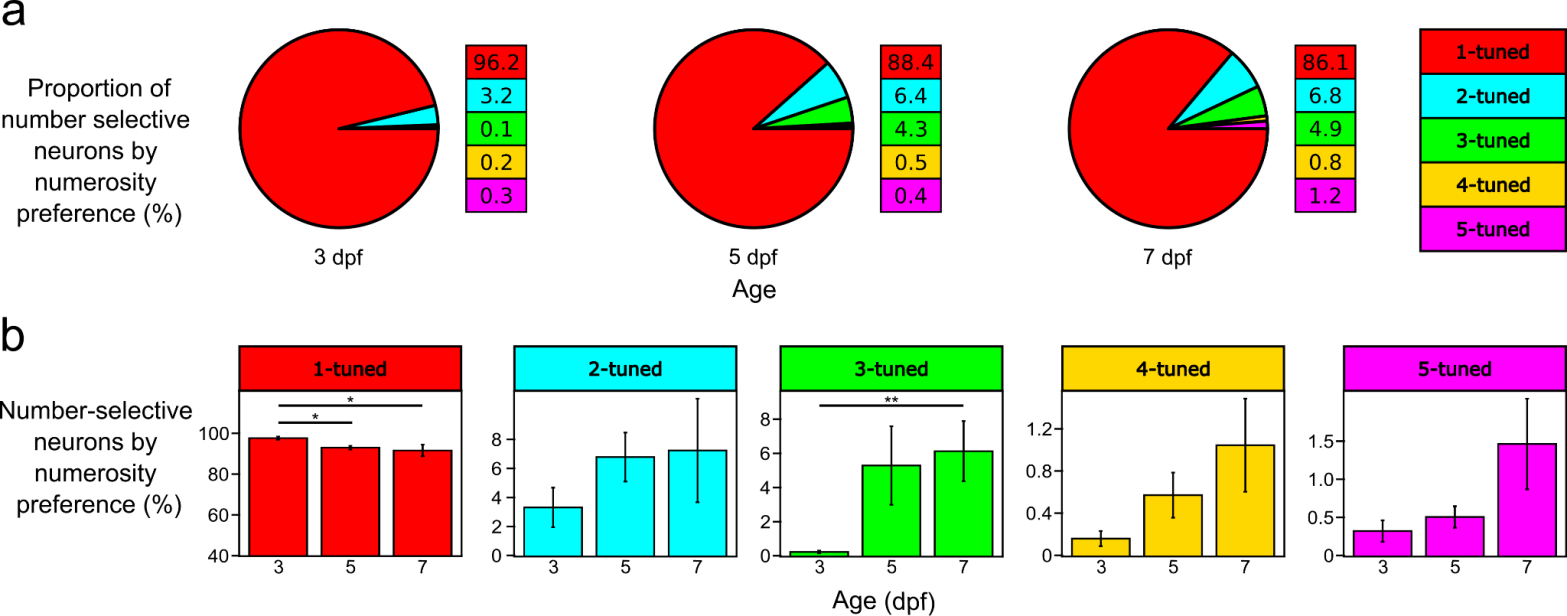
Populations of neurons tuned to specific numerosities show redistribution of number preference during early development. **a** Proportion of number-selective neurons as a percentage of all detected number-selective neurons across 3, 5, and 7 dpf. 1-tuned neurons = red, 2-tuned neurons = cyan, 3-tuned neurons = green, 4-tuned neurons = yellow, 5-tuned neurons = magenta. n = 5. **b** Percentages of number-selective neurons preferring specific numerosities for 3-, 5-, and 7 dpf. Percentage is expressed as a proportion to all detected number-selective neurons. Pairwise comparisons were performed using a Mann-Whitney U-test for each numerosity, multiple comparisons were adjusted using a Bonferroni correction (alpha = 0.016). Error bars = SEM, n = 5, * = p < 0.05, ** = p < 0.01.

As the zebrafish larval age increases across 3, 5, and 7 dpf, we found that the proportion of neurons tuned to numerosities of two or more shows a trending increase with age (3dpf:4%; 5dpf:12%; 7dpf:14%) (Figure 3a). When comparing the tuning preferences of the number-selective neurons (Figure 3b), we found a significant decrease of 1-tuned neurons in 3 dpf (96 ± 1%) compared to 5 (88 ± 2%) and 7 (86 ± 5%) dpf groups (p < 0.05). For 3-tuned neurons, we found a significant increase in 7 dpf (5 ± 1%) compared to the 3 dpf (0.2 ± 0.1%) group (p < 0.01).

### Number-selective neurons primarily localize to the forebrain and midbrain

**Figure 4.**
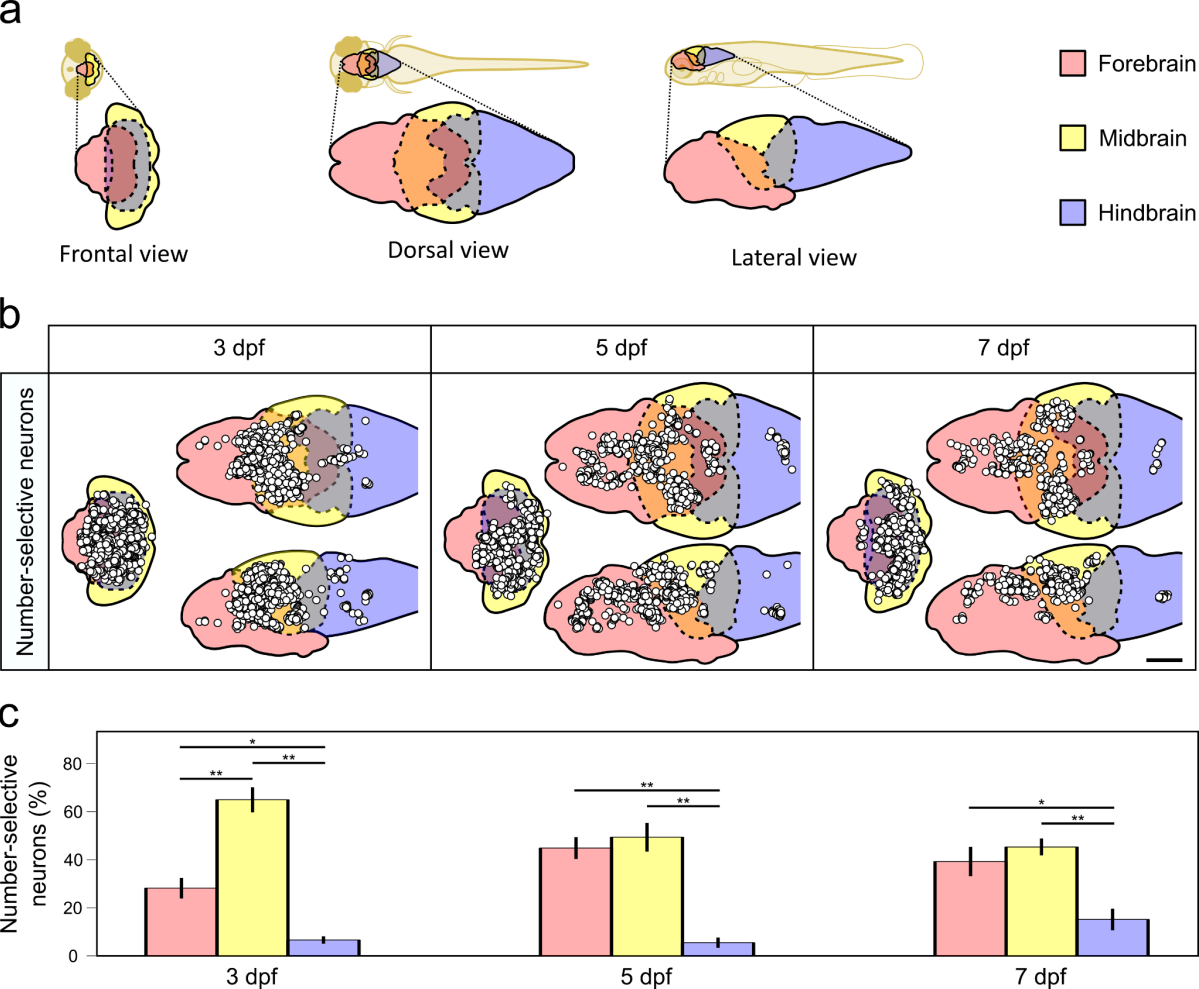
Number-selective neurons are primarily detected in the forebrain and midbrain. **a** The 3D map of the brain was divided into three major brain regions (forebrain, midbrain, hindbrain). Solid lines indicate delineation of major brain regions, dash lines indicate overlapping regions. **b** Locations of number-selective neurons in three different individual larval zebrafish at three stages of development, representing the results as point maps in orthographic projections. The white circles represent the centers of each identified number-selective neuron. Columns indicate age. Scale bar: 100 μm. **c** Comparison of number-selective neuron distribution across brain regions of three stages of development. Number-selective neurons per region are normalized by the total number of number-selective neurons detected in the whole brain. Comparisons were performed using a two-way ANOVA for age and brain region followed by Tukey’s HSD. Error bars = SEM, n = 5, * = p < 0.05, ** = p < 0.01.

Number-selective neurons were detected across the forebrain, midbrain, and hindbrain of 3, 5, and 7 dpf groups. To show the locations of the number-selective neurons, we registered all imaged samples onto the respective brain templates of each age group (see Methods) and mapped out the centroids of all identified neuronal nuclei (Figure 4a–b, Supplementary Figure 5). We found a majority of number-selective neurons were localized to the forebrain and midbrain (Figure 4c). For all three age groups, the forebrain (3dpf:28 ± 4%; 5dpf:45 ± 5%; 7dpf:39 ± 6%) and midbrain (3dpf: 65 ± 5%; 5dpf: 49 ± 6%; 7dpf: 45 ± 4%) had proportionally more number-selective neurons than the hindbrain (3dpf: 7 ± 2%; 5dpf: 6 ± 2%; 7dpf: 15 ± 5%) (p ≤ 0.01). We did not identify any apparent mapping based on preferred numerosities (Supplementary Figure 6). In the 3 dpf group, we found significantly less neurons in the forebrain than the midbrain (p < 0.001).

Building upon these findings, we further investigated the subregions of the forebrain (eminentia thalami, hypothalamus, pallium, pretectum, thalamus, subpallium) and found no significant changes with age (Supplementary Figure 7, Supplementary Table 6).

### Number stimulus can be decoded from number-selective neurons

**Figure 5.**
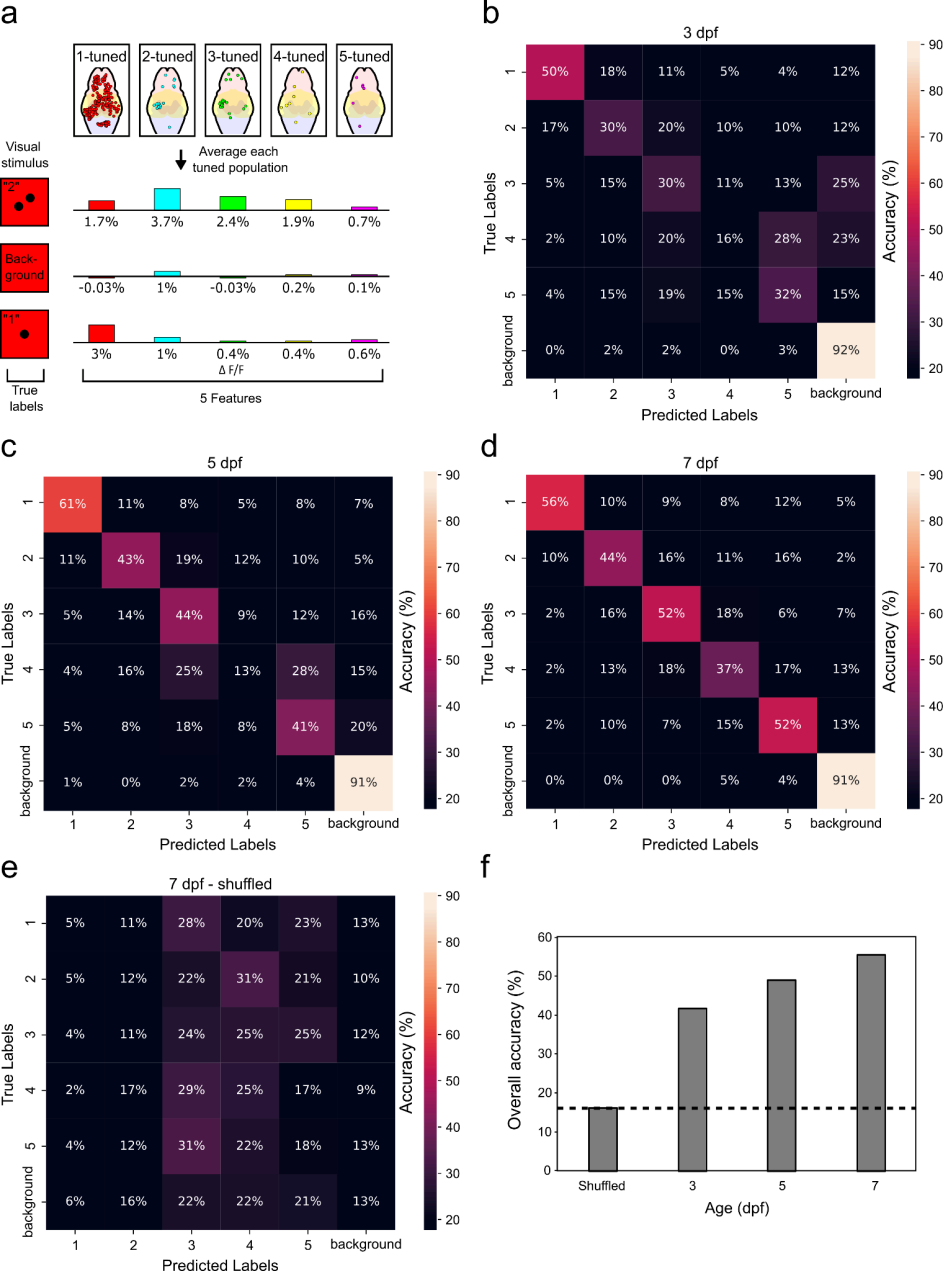
Prediction accuracy of the numerical stimuli from Ca^2+^ activity using SVM classifier shows increased performance with age. **a** Feature extraction of Ca^2+^ activity from numerically-tuned neurons. For each training and testing instance, the average Ca^2+^ activity of each neuron population tuned to the 5 numerosities was calculated and shown as a percentage of ΔF/F (5 input features). The visual stimulus (1, 2, 3, 4, 5, background/no dot) serves as the true label for each instance. Each sample larval fish is comprised of 288 instances (48 repetition * 6 visual stimulus type). Cross validation was performed using a leave-one-out cross validation where training was performed on the data from 4 of 5 larval fish and tested on the excluded sample, then repeated on a different excluded sample. **b, c, d, e** Confusion matrix of the support vector machine (SVM) classifier of 3, 5, and 7 dpf groups and a shuffled 7 dpf group. The percentage indicates the number of predictions out of the total instances of each true label. Random chance = 16.7%. **f** Overall SVM classification accuracies for the three age groups. Dash line indicates chance level, calculated as the accuracy of shuffled data from the 7 dpf group.

To determine if the Ca^2+^ activity of number-selective neurons across the brain is sufficient to predict the correct number of dots shown during a visual stimulus, we trained a support vector machine (SVM) supervised classifier to estimate the visual stimulus from the recorded activities (Kirschhock & Nieder, 2022). The features were extracted averaging the Ca^2+^ activity of all identified number-selective neurons for each preferred numerosity (Figure 5a, Methods). The Ca^2+^ activity averages for number preferences 1–5 serve as the five input features and are used to predict six types of stimuli (1, 2, 3, 4, 5 dot, no dot). The classifier was trained on four out of five individual zebrafish in each age group and tested on the remaining one, enabling generalized testing across conspecifics.

To assess the performance of the SVM classifier, we used a confusion matrix to evaluate the classifier accuracy (Figure 5b, c, d, e). The confusion matrix shows the prediction instances (columns) during a visual numerical dot stimulus or “true label” (rows). Each entry of the matrix’s main diagonal shows correct prediction of the true labels. By averaging the diagonal entries, the overall classifier accuracy can be obtained. At 3 dpf, the prediction accuracy was above chance level (16.7%) for the numerosities 1 (50%), 2 (30%), 3 (30%), and 5 (32%) (Figure 5b). At 5 dpf, the general is similar but with an increase in accuracy: 1 (61%), 2 (43%), 3 (44%), and 5 (41%) (Figure 5c). At 7 dpf, for numerosities greater than 2 we see a general increase in accuracy: 1 (56%), 2 (44%), 3 (52%), 4 (37%), and 5 (52%) (Figure 5c). The prediction improvement is confirmed by the overall classifier accuracy increasing with age (42%, 48%, 55% for 3, 5, and 7 dpf, respectively) (Figure 5f, Supplementary Table 7).

### Ethanol inhibits number-selective neurons in the forebrain

**Figure 6.**
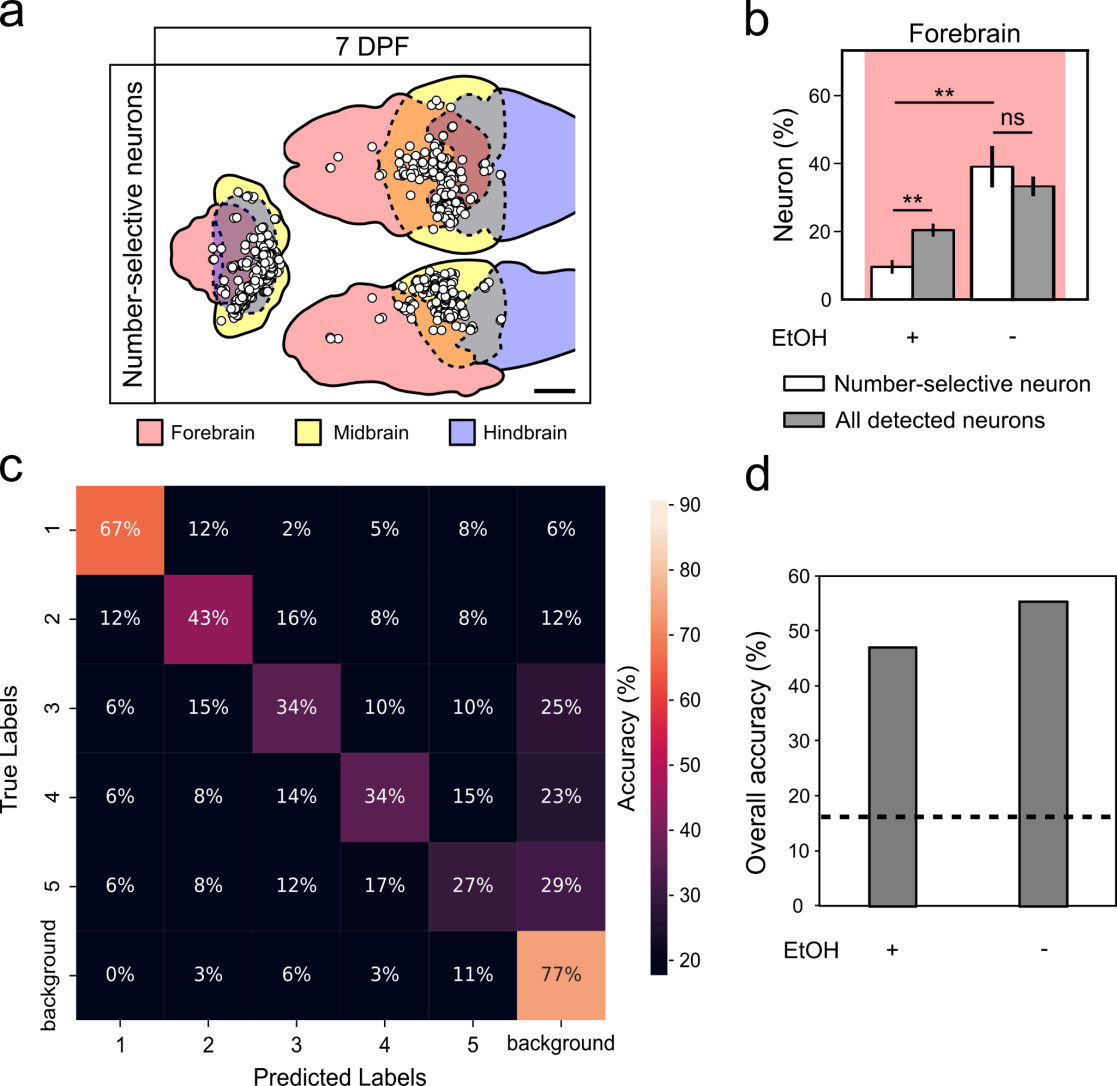
Ethanol alters the activity of number-selective neurons in the forebrain. **a** Location of number-selective neurons in three different brain regions in a single 7 dpf larval zebrafish treated with ethanol. Refer to the caption for Figure 4b for a detailed description. **b** Percentage of number-selective neurons in the forebrain during ethanol treatment. Number-selective and active neurons in the forebrain are normalized to all number-selective or active neurons (respectively) across the whole brain. Pairwise comparisons were performed using a Mann-Whitney U-test with a Bonferroni correction for multiple comparisons (alpha = 0.17). Error bars represents SEM, n = 5, ** denotes p < 0.01. **c** Confusion matrix of the SVM classifier of the numerical stimuli using Ca^2+^ activity during ethanol treatment. Refer to the caption for Figure 5b, c, d, e for a detailed description. **d** Overall SVM classification accuracies for the 7 dpf EtOH treatment groups. Dash line indicates chance level, calculated as the accuracy of shuffled data from the 7 dpf untreated group.

Acute ethanol exposure affects learning and memory processing in zebrafish (Sartori et al., 2022). To understand how this affects number-selective neurons we examined the effect of ethanol on number-selective neurons of 7 dpf larvae compared to the untreated larvae. Location of detected number-selective neurons in the forebrain is noticeably less when treated with 1.5% ethanol (Figure 6a). The percentage of number-selective neurons in the ethanol-treated group (10%) showed a significant decrease in the forebrain when compared to detected active neurons in the forebrain brain (20%) and the forebrain of untreated group (39%) (n=5 for each treatment group) (Figure 6b, Supplementary Figure 8). To measure the predictive capacity of number-selective neurons of an ethanol-treated group, we trained a supervised classifier to predict the visual stimulus using the Ca^2+^ activity (Figure 6c). The overall accuracy of the ethanol-treated group (41%) was decreased compared to the untreated group (55%) (Figure 6d). This effect is mainly driven by the decreased accuracy when predicting numerosities of 3, 4, and 5. These results suggest ethanol may impair the function of number-selective neurons.

## Discussion

In this study, we investigated the neural basis of number sense in larval zebrafish, focusing on the tuning of neurons to specific visual-based numerosities under different conditions. Our work has, for the first time, discovered the existence of number-selective neurons in larval zebrafish. The fast volumetric imaging rate of one volume per second and the high signal-to-noise ratio of light sheet microscopy enabled whole-brain recording and segmentation of approximately 17,500 active neurons per larva. The use of two-photon excitation allowed for increased depth of coverage (deeper imaging) and reduced visual artifacts compared to one-photon excitation (Truong et al., 2011; Vito et al., 2022; Wolf et al., 2015).

Consistent with studies using chick models (Kobylkov et al., 2022) and human infants (Izard et al., 2009; Xu & Spelke, 2000), we detected number-selective neurons during early post-embryonic (equivalent to post-natal) stages in zebrafish. Notably, the existence of these number-selective neurons at 3 dpf precedes any known numerically-driven behaviors such as hunting and shoaling (which start at 5 and ~24 dpf, respectively) (Adam et al., 2024; Borla et al., 2002; Lucon-Xiccato et al., 2023; Sheardown et al., 2022a), underscoring the fundamental role and necessity of early numerical cognition for survival.

Consistent with studies using chick models (Kobylkov et al., 2022) and human infants (Izard et al., 2009; Xu & Spelke, 2000), we detected number-selective neurons during early post-embryonic (equivalent to post-natal) stages in zebrafish. Notably, we observed these neurons at 3 dpf, which is before the onset of known numerically-driven behaviors that typically begin at 7 dpf (Adam et al., 2024; Lucon-Xiccato et al., 2023). This finding suggests that the development of these neurons precedes and potentially facilitates these behaviors, highlighting the critical importance of early numerical cognition for survival.

The proportion of neurons tuned to numerosities of two or more shows a trending increase with age (Figure 3a). A significantly increased proportion of neurons preferring 3 objects was detected after 3 dpf (Figure 3b). These results suggest number-selective neurons develop in an ordinal fashion with age. An interesting question for future studies is whether this increase is due to the generation of new neurons preferring higher numerosities or the re-tuning of existing neurons. This could be resolved by application and further refinement of our experimental platform to observe number-selective neurons in the same zebrafish longitudinally over development time.

The increased proportion of neurons preferring larger numerosities (>2) developing after 3 dpf may be caused by an improvement in visual acuity rather than changes to number-selective neurons. However, the zebrafish eye is emmetropic at 3 dpf (Easter & Nicola, 1996), and no differences in visual acuity were detected when comparing larvae at 4, 5, and 6 dpf (Haug et al., 2010). Furthermore, recognition of 5 dots does not require finer visual acuity than 2 dots when maintaining equivalent inter-distances (Figure 1c). Because we detected neurons preferring 2 dots in 3 dpf larvae, the increase of neurons preferring larger numerosities in older larvae is unlikely to be caused by improved visual acuity.

We identified number-selective neurons localized throughout the forebrain and midbrain (Figure 4b). In the 3 dpf group, we found significantly less neurons in the forebrain compared to the midbrain, whereas in the 5 and 7 dpf groups, the forebrain contained a similar proportion of these neurons (Figure 4c), likely due to the forebrain being more developed in the older fish (Cheng et al., 2014). Further analysis of the forebrain subregions found no significant age-related changes in (Supplementary Figure 7, Supplementary Table 6). These results suggest that while overall development affects neuron distribution, the specific sub regional changes may not happen until the brain matures beyond 7 dpf.

In the mammalian brain, most number processing is to our knowledge localized to the prefrontal and parietal cortices (Nieder et al., 2002; Piazza et al., 2004). In non-mammals, such as zebrafish, the pallium generally fulfills the role of the prefrontal cortex (Medina et al., 2019). However, the non-mammalian brain lacks a structure that is directly analogous to the parietal lobe; instead, the optic tectum of the midbrain serves many cortical functions such as sensory processing and spatial perception (Förster et al., 2020; Gazzola et al., 2018; Muto et al., 2013). In line with our result of finding number-selective neurons in the midbrain, emerging studies suggest subcortical and optic tectal involvement in visuospatial processing on numerosities (Bengochea et al., 2023; Bengochea & Hassan, 2023; Collins et al., 2017; Lorenzi et al., 2021). This suggests that the optic tectum participates in more complex functions than its long-studied roles in visual mapping and sensory integration.

To assess the predictive capabilities of the number-selective neurons across 3, 5, and 7 dpf zebrafish, we trained a supervised classifier using their underlying Ca^2+^ activity. The prediction accuracy of the classifier increases with age for 2–5 objects, indicating that higher number-selective neurons become more specific (generating more action potentials in response to specific numerical stimuli) as the larval zebrafish matures. Similar to the findings of number sense in crows (Kirschhock & Nieder, 2022), our results show that most misclassifications occurred near the correct choice, suggesting a numerical distance effect (i.e. discrimination errors arise between closer numerosities) (Moyer & Landauer, 1967; Nieder, 2011). From 3 to 5 dpf, the average prediction accuracy of 2, 3, and 5 objects increased from ~30% to ~43%, but interestingly the accuracy of 4 objects remained near random chance (17%) until 7 dpf.

A plausible explanation for the decreased accuracy of 4 objects for 5 dpf fish is that numerosity of 4 represents a transition point between Object Tracking System/Parallel Individuation System (OTS/PIS) and Approximate Number System (ANS) (Feigenson et al., 2004; Hyde, 2011; Sheardown et al., 2022b). The OTS/PIS is thought to be responsible for tracking and representing small quantities of objects with high precision, typically up to four items. Whereas the ANS operates on an approximate level, allowing for rapid estimation of small (<4) and large (>4) numerical magnitudes beyond the capacity of the OTS/PIS. If the OTS develops ordinally then neurons preferring 4 objects would develop last whereas neurons preferring 5 objects would have emerged earlier with the ANS.

When larval zebrafish were exposed to ethanol, the activity of number-selective neurons decreased in the forebrain (Figure 6a–b). Given ethanol’s well-known propensity to inhibit information processing in the frontal lobe of humans (Koelega, 1995; Tzambazis & Stough, 2000), it is likely that the number-selective neurons in the forebrain, which are implicated in complex higher-order functions such as learning and memory (Dempsey et al., 2022; Rodríguez et al., 2002), are selectively affected. When predicting the stimulus from the Ca^2+^ activity using an SVM classifier, the overall accuracy decreases for larger numerosities (>2) (Figure 6c). Interestingly, the prediction accuracy of 1 dot showed improvement. One possible explanation is ethanol treatment is inhibiting the number-selective neurons part of the ANS system that prefer 1, leaving OTS neurons remaining which have a more precise representation of numbers (Feigenson et al., 2004).

Our findings contribute to the growing understanding of the developmental stages of cognitive abilities in vertebrates, offering new insights into how early neural circuits involved in numerosity evolve even before behaviorally measurable traits emerge. The identification of number-selective neurons in larval zebrafish as early as 3 dpf, well before the onset of numerically-driven behaviors, emphasizes the critical role of early neural development in the establishment of cognitive functions necessary for survival. This study not only adds to the foundational knowledge of numerical cognition in non-mammalian species but also opens avenues for comparative studies across vertebrate models, including primates and humans, to explore the evolutionary conservation of these neural circuits.

## Methods

### Animal care

Casper zebrafish (*Danio rerio*) expressing a pan-neural, nuclear-localized fluorescence Ca^2+^ reporter (elavl3:H2B::jGCaMP7f) was a gift from the lab of David Prober at California Institute of Technology. Larval fish were raised accordingly to establish methods (Avdesh et al., 2012) with modifications: 13:11 hr (light:dark) and fed dry food twice daily after 5 days post-fertilization (dpf). Experiments used zebrafish ranging from 3–7 dpf. Sex is not defined at this stage of development. Larvae were raised in 50 mL petri dishes with approximately 50 larvae per dish. E3 medium (5 mM NaCl, 0.17 mM KCl, 0.33 mM CaCl2, 0.33 mM MgSO4). All animal procedures conformed to the institutional guidelines set by the University of Southern California Department of Animal Research.

### Calcium imaging

Zebrafish larvae were embedded in 2% low-melting-point agarose (Invitrogen cat 16520100) and mounted in a custom sample holder. During image acquisition, the larvae were perfused with oxygenated water using a peristaltic pump and heated to 28C. Image acquisition was performed on a custom-built microscope (Keomanee-Dizon et al., 2020) that was further modified by optimizing the polarization and additional laser pulsing to increase the fluorescence signal (Luu et al., 2024; Vito et al., 2020, 2022). The sample was imaged via two-photon excitation using a Chameleon Ultra II Ti:Sapphire laser (Coherent) at 920 nm with approximately 300 mW peak power and 180 mW average power (combined excitation laser at the sample after splitting). Emitted light was bandpass filtered at 525 ± 45 nm and collected using a 20× 1.0 NA water dipping objective (Olympus). Continuous images were acquired at a rate of 1 second per volume, in which a volume is composed of 60 z-slices at 540 × 296 pixels across 230 μm (~900 × 500 × 230 μm3, equating to a 3.83-μm-thick section) at a pixel resolution of 1.68 μm. The acquisition time per sample is approximately 90 minutes excluding a 30-minute acclimation period totaling ~100gb of data. Software control and hardware synchronization for image acquisition was performed as previously described (Keomanee-Dizon et al., 2020) using μManager (Edelstein et al., 2010b) and LabVIEW.

### Stimuli Generation

Dot patterns were generated using GeNEsIS (Zanon et al., 2022) and controlled for convex hull, inter-distance, total area, total perimeter, and radius in (Figure 1b). Convex hull describes the smallest convex polygon that encloses all of the elements, inter-distance is the average distance between the elements. Total area equates average brightness and cumulative surface area for different numerosities. Total perimeter equates the cumulative circumference of all the elements for different numerosities. The parameters are summarized in Supplementary Table 8. Note: parameter values apply by use case (“1” dot stimuli does not have convex hull or inter-distance parameters, constant radius does not use radius variability). Angular diameter of dots were kept above 5° to maintain visibility (Haug et al., 2010) and below 18° to minimize an escape response (Temizer et al., 2015). Numerical elements were colored black on a red background to simulate objects’ contrast in the natural environment and to prevent disassembly of the photoreceptor of the photoreceptor (Emran & Dowling, 2010).

### Visual number-based display

Visual stimuli were projected onto a diffuser placed 19 mm away from the larvae (figure 1a). The diffuser is made of cellulose acetate (Scotch Magic tape) that faces the right eye of the larval fish and is placed orthogonally to its body axis. Illumination was generated using a Qumi Q5 LED Projector (Vivitek) and bandpass filtered at 660 ± 45 nm (Thorlabs).

Each numerical stimulus is presented 48 times following a pseudo-random order. The stimulus is displayed for 1 second followed by varying inter-stimulus intervals of a blank red background between 15–30 seconds. The display area is 22 mm in diameter or 66° in angular diameter. Stimulus control was performed using the PsychoPy toolkit (Peirce et al., 2019).

### Ethanol administration

To determine the appropriate ethanol concentration, we adapted methods from previous studies (Dlugos & Rabin, 2003; Vossen et al., 2022) to assess larval zebrafish swimming behavior and mortality after treatment. In triplicates, five 7 dpf zebrafish were immersed in 15 mm × 100 mm Petri dishes containing 1, 1.5, and 2% ethanol in E3 media for 1.5 hours (image acquisition duration). We then screened for hyperactivity by gently tapping on the Petri dish and chose the 1.5% ethanol concentration for this study. Before image acquisition, the zebrafish were treated with 1.5% ethanol 30 minutes prior and then continuously perfused with oxygenated E3 media containing 1.5% ethanol during imaging.

### Cell segmentation

The datasets were first motion corrected using Advanced Normalization Tools (Avants et al., 2009). Cell segmentation was performed using a python library designed for calcium imaging (CaImAn) (Giovannucci et al., 2019). CaImAn consists of a series of functions enabling the separation of neurons using Ca^2+^ activity in time and space by applying non-negative matrix factorization. Prior to cell segmentation, the data size was reduced by selecting only the time points around the stimulus presentation (3 s before stimulus + 1 s stimulus + 5 s post-stimulus; see Figure 2). The final volumetric time series was reduced from 5,472 s to 2160 s (9 s window X 5 numerosities X 48 repetitions). Large data handling and annotation were managed using an inhouse python library that facilitated image processing (Nadtochiy et al., 2023).

The segmentation was performed in 2D where each time point consisted of 60 z-slices with the following parameters: ‘decay_time’ = 5 (length of a typical transient in seconds); ‘gSig’ = 3×3 (expected half size of neurons in pixels); min_SNR = 1.5 (signal to noise ratio to accept a component); rval_thr = 0.85 (space correlation threshold to accept a component). The ‘K’ parameter is the expected number of cells to be segmented that serves as a starting point for optimization. Since the number of cells expected in each z-slice varies, the ‘K’ parameter is estimated based on the standard deviation of Ca^2+^ flux in each z-slice over time. The standard deviation image is thresholded (min_std + 0.08*(max_std - min_std)) resulting in an image with pixels that represent cells with Ca^2+^ flux. The maximum number of the resulting pixels are then divided by ‘gSig’ to approximate the number of cells per z-slice. Since a single cell’s Ca^2+^ signal can span across 2–3 z-slices, we eliminate duplicates by merging cells with both a centroid distance less than 1 pixel and with a Ca^2+^ activity correlation coefficient higher than 0.95.

### Number neuron selection

Identification of numerically-tuned neurons involved additional preprocessing steps that removed camera shot noise and established a baseline fluorescence. To remove false positive segmented cells caused by the camera shot noise (identified as segmented cells that were found outside of the brain), we calculated the coefficient of variation (CV), the ratio of the standard deviation to the mean, for each timepoint in the peristimulus windows. We found a CV of 0.05 was sufficient to remove false positive cells related to shot noise. Baseline fluorescence (F_0_) was defined as the average of three time points before the visual stimulus around the stimulus presentation (peristimulus window) for each numerosity.

To differentiate neurons responsive to numerosity from those responsive to nonnumerical covariables (size and spread), we utilized a two-way permutation ANOVA. This involved randomizing the labels associated with the data and calculating the F-value. We repeated this process 10,000 times to construct a null distribution based on simulated F-values to which the actual F-value was compared to get the p-value. The criteria for identifying a number-selective neuron must have a significant main effect for the numerical stimulus (alpha = 0.01), a non-significant main effect for the nonnumerical covariables, and no interaction effect.

### Brain spatial registration and region segmentation

All samples were first registered to a brain template of each respective age group with ITKsnap (Yushkevich et al., 2006) using the average Ca^2+^ signal in time. All identified neuron centers were remapped to the final brain template to compare across different fish. To identify subregions of the forebrain, we registered the brain templates to the mapZebrain atlas (Kunst et al., 2019) using affine transformation, then selected the available subregion Boolean masks.

### Supervised classification

To test the predictive properties of the number-selective neurons, we applied a support vector machine (SVM) based supervised classifier using a linear kernel. The classifier used the underlying Ca^2+^ activity to predict the visual number-based stimulus. To extract the features, we calculated the average Ca^2+^ activity of the neurons tuned to each of the five numerosities during a 2-second window encompassing the 1-second visual stimulus and the following post-stimulus second. These five average activities served as input features for the SVM. The six classes (true labels) consisted of the five numerosities (1–5 objects from the visual stimulus) and the average Ca^2+^ activity during the frames preceding the stimulus representing the no stimulus or background baseline.

We trained the SVM model on each experimental group (3 dpf, 5 dpf, 7 dpf, 7 dpf + EtOH) to classify trials based on the five extracted features. We applied a leave-one-out cross-validation scheme, where the model was trained on data from four fish within a group and tested on the remaining fish. This procedure was repeated five times, each time excluding a different fish. The final confusion matrices (Figures 4b–d, and 5c) were obtained by combining the test results from all five repetitions.

### Statistical analysis

Statistical analyses and graph preparation were conducted using: custom python scripts, seaborn library (Waskom, 2021), Inkscape.

## Supplementary Material

Supplement 1

## Figures and Tables

**Key resources table:** 

Reagent type (species) or resource	Designation	Source or reference	Identifiers
Genetic reagent (*Danio rerio*)	Zebrafish: Tg(elavl3:H2B::jGCa MP7f)	(Dana et al., 2019b; Yang et al., 2022), gift from David Prober	RRID:Addgene_104488
Python Library	Analysis tools: ANTs	(Avants et al., 2009)	https://github.com/ANTsX/ANTs
Python library	Analysis tools: NuMan		https://github.com/LemonJust/numan
Python library	Analysis tools: numan_plus		https://github.com/MirkoZanon/numan_plus
Python library	Analysis tool: seaborn	(Waskom, 2021)	https://seaborn.pydata.org/index.html
Python library	Data management: VoDEx	(Nadtochiy et al., 2023)	https://github.com/LemonJust/vodex
Python library	Cell Segmentation	(Giovannucci et al., 2019)	https://github.com/flatironinstitute/CalmAn
Python library	Stimuli presentation: PsychoPy	(Peirce et al., 2019)	https://psychopy.org/index.html
Software/Python Library	Image analysis toolkit: ITK-SNAP	(Yushkevich et al., 2006)	http://www.itksnap.org/
Software	Microscope GUI, μManager	(Edelstein et al., 2010a)	https://doi.org/10.1002/0471142727.mb1420s92
Software	Stimuli generation: GeNEsIS	(Zanon et al., 2022)	https://github.com/MirkoZanon/GeNEsIS

## Significance

- Numerically responsive neurons are detected by 3 days post-fertilization (dpf) in zebrafish
  - Number-selective neurons preferring quantities greater than one increases after 3 dpf
- Neurons that are highly responsive to specific numbers of objects are mainly localized to the forebrain and midbrain
- Number stimulus can be decoded from Ca^2+^ activity and show increased performance with age
- Administration of ethanol decreases the activity of number-selective neurons in the forebrain
