# Supplementary material for: Neural Basis of Number Sense in Larval Zebrafish
Source: bioRxiv. 2024 Sep 5:2024.08.30.610552. Preprint. [Version 2] doi: 10.1101/2024.08.30.610552 (PMC11406567; doi:10.1101/2024.08.30.610552)
Supplement: Supplement 1 [file media-1.pdf]

Supplementals

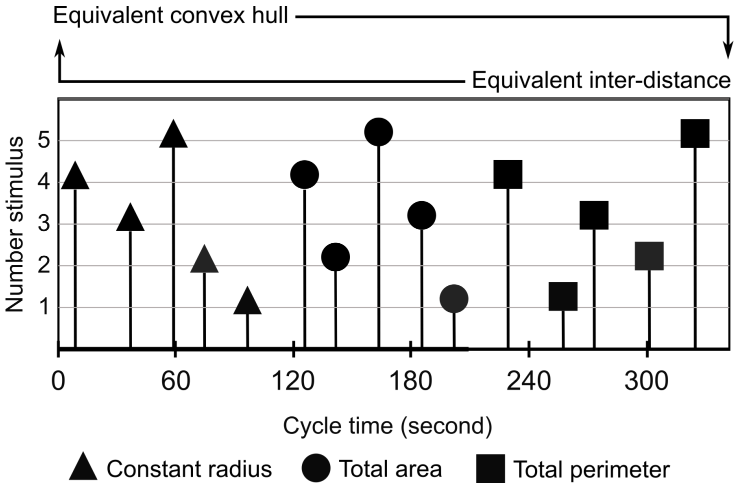

Supplementary Figure 1. Sequence of stimuli including all possible combinations of spread and sizes using a new pattern for each stimulus.

Each stimulus lasts for 1 second, and the inter-stimulus duration varies between 15 and 27 seconds. A stimulus cycle is 684 seconds in total when including both convex hull and inter-distance controls. When a pseudo-random cycle is repeated, a novel dot pattern is displayed. The cycle is repeated 8 times per sample.

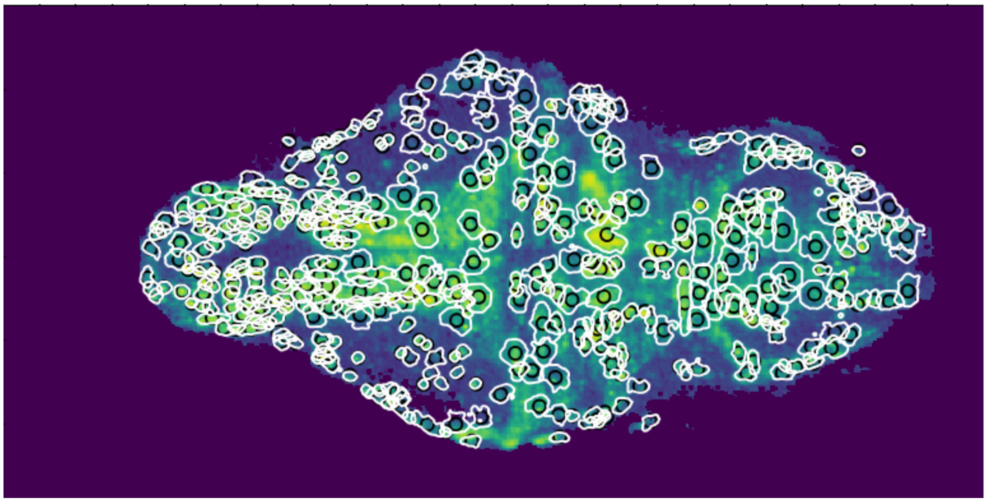

Supplementary Figure 2. Segmentation output using CaImAn toolbox.

Black circles indicate neuron centers. White boundaries indicate segmented Ca^{2+} signal.

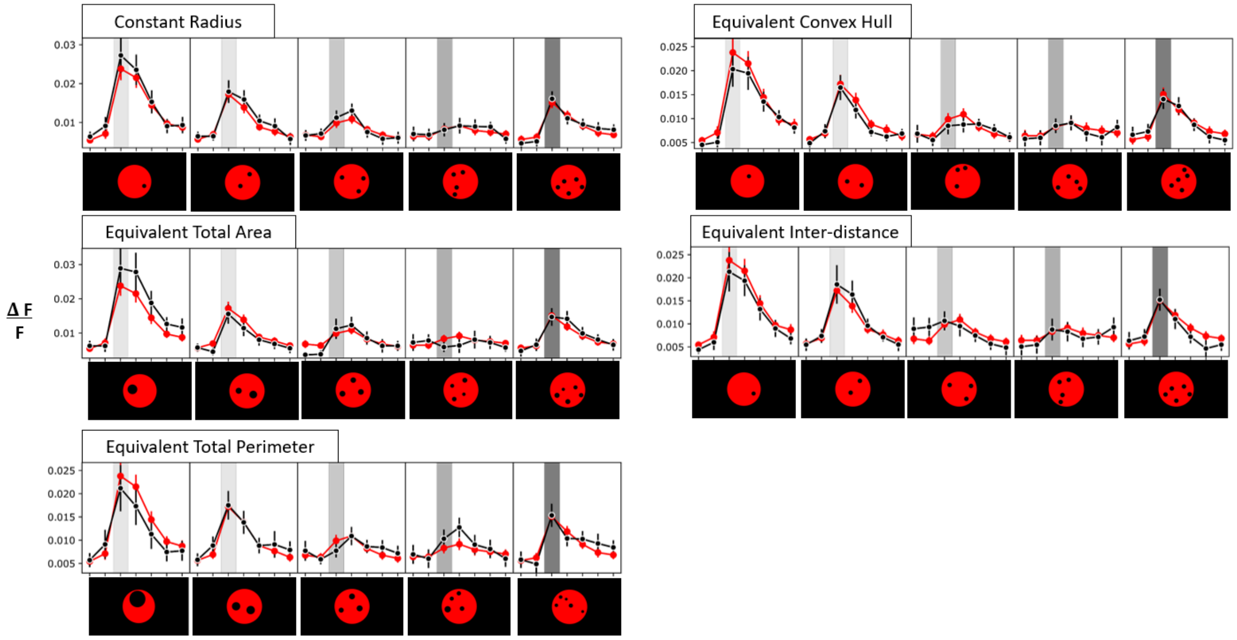

Supplementary Figure 3. Example traces of geometric controls of the number-based dot stimuli

Ca^{2+} signal traces from a number-selective neuron which responds to changes in number rather than geometric variations (covariates). Black lines indicate the mean Ca^{2+} signal during any stimuli presentation, while red lines indicate the mean Ca^{2+} signal specific to the geometric covariates. Gray bars indicate stimulus onset, tick marks represent seconds, error bars indicate the standard error of the mean (SEM).

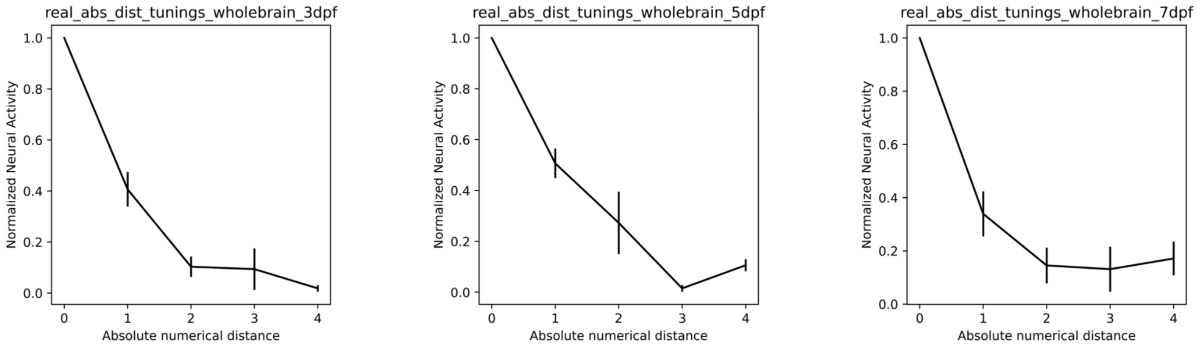

Supplementary Figure 4. Tuning curve of 3, 5, and 7 dpf age groups

Overall tuning curves, averaged across five fish per age group, represent the normalized average Ca^{2+} activity of all number-selective neurons in response to stimuli at different numerical distances from the preferred numerosity. The response of a neuron to its preferred numerosity is represented by 0. See Supplementary Table 1 and 2 for neuron counts.

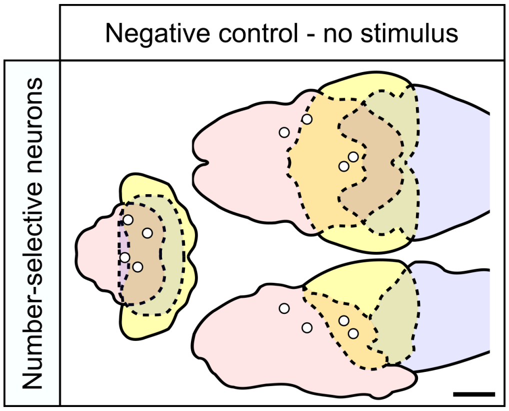

Supplementary Figure 5. No-stimulus negative control

Locations of number-selective neurons in one larval zebrafish as orthographic projections without presenting number stimuli. The white circles represent the centers of each identified number-selective neuron. Scale bar: 100 µm.

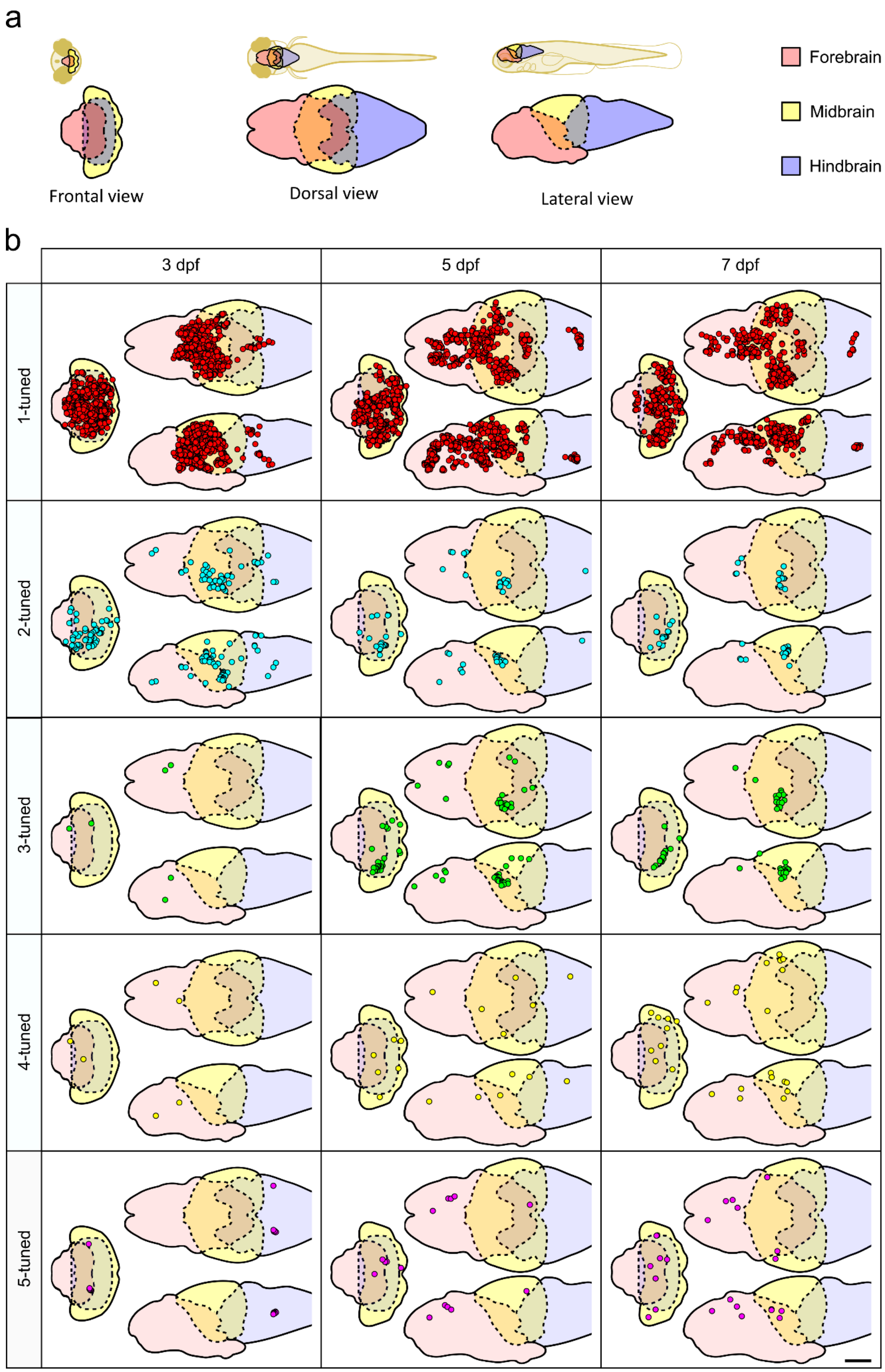
a
Frontal view
Dorsal view
Lateral view
Forebrain
Midbrain
Hindbrain
b
3 dpf
5 dpf
7 dpf
1-tuned
2-tuned
3-tuned
4-tuned
5-tuned

Supplementary Figure 6. Localization of number-selective neurons at three stages of development

a The 3D map of the brain was divided into three major brain regions (forebrain, midbrain, hindbrain). Solid lines indicate delineation of major brain regions, dash lines indicate overlapping regions.

b Locations of number-selective neurons in three different individual larval zebrafish at three stages of development, representing the results as point maps in orthographic projections. The individual-colored dots represent the centers of each number-selective neuron extracted using the pipeline presented in Figure 2a. Columns indicate age; neurons responding with specific number tunings are shown as rows. Scale bar: 100 μm.

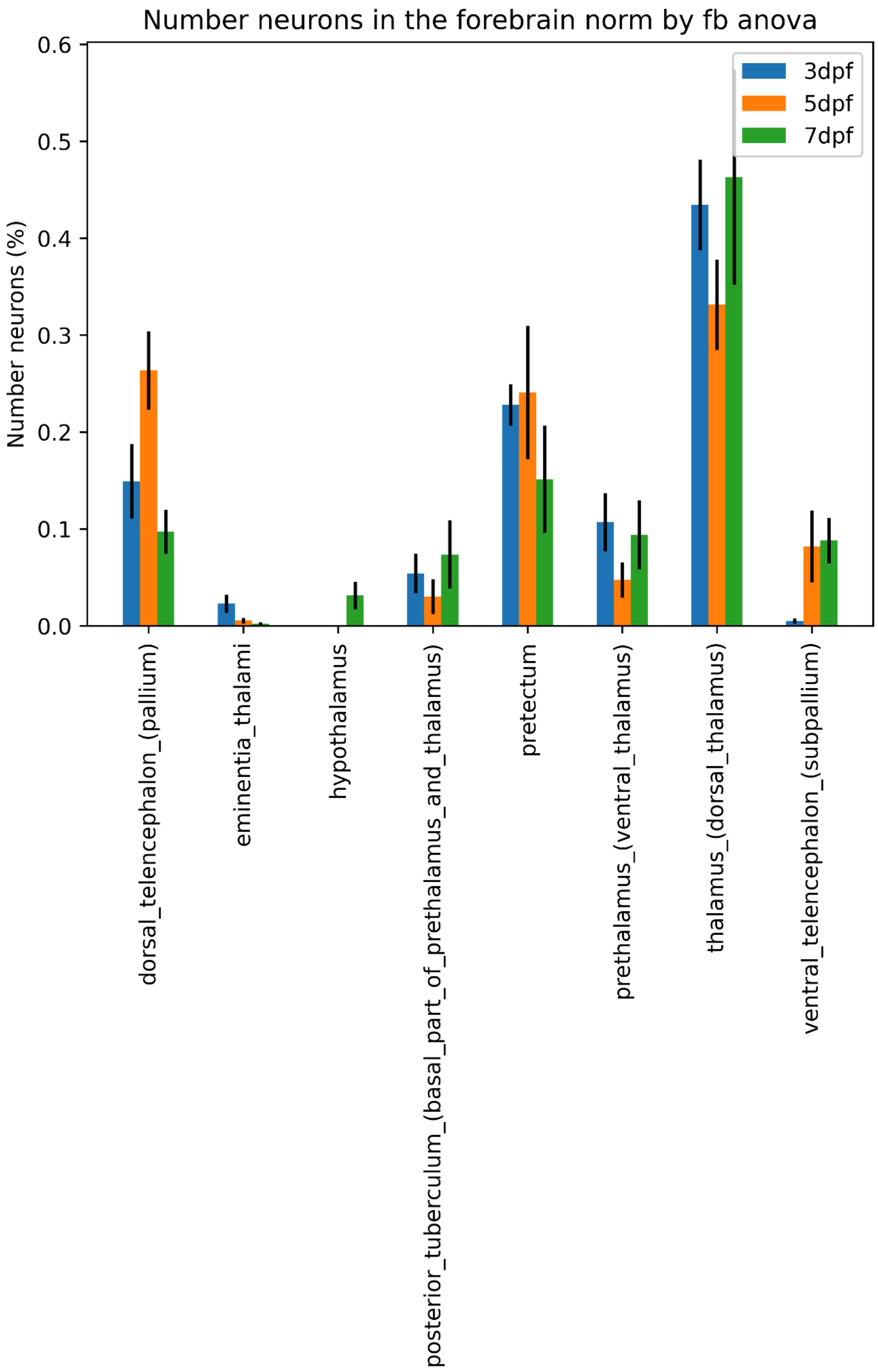

Supplementary Figure 7. Distribution of sub-regional number-selective neurons normalized by total number-selective neurons in the forebrain.

See Supplementary Table 6 for p-values and f-scores.

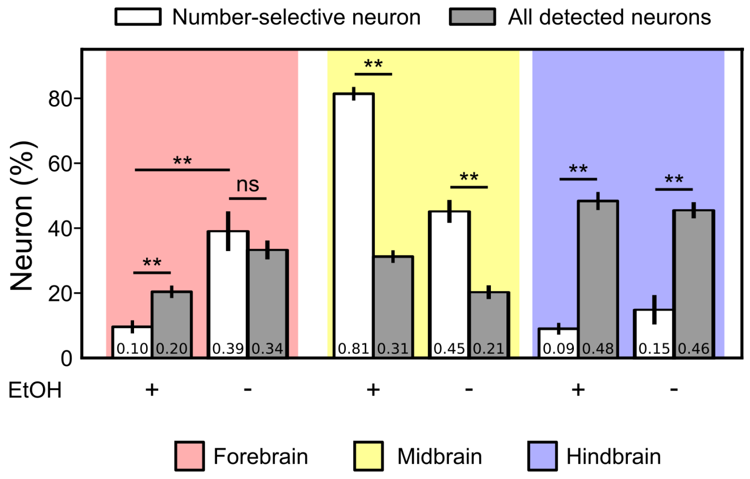

Supplementary Figure 8. Distribution on number-selective and all active neurons across three major brain regions during ethanol administration.

Comparison of number-selective neuron distribution across brain regions between ethanol groups. Number-selective neurons per region is normalized by the total number of number-selective neurons detected (white bar) or all detected neurons (gray bar). Pairwise comparisons were performed using a Mann-Whitney U-test with a Bonferroni correction for multiple comparisons (alpha = 0.17). Error bars represents SEM, n = 5, ** denotes $p < 0.01$.

Supplementary Table 1. Group averages of identified neurons.

Negative control group underwent normal acquisition protocol with only a visible red background and no dot stimulus.

	Number-selective neurons		Active neurons	
dpf	Average	SEM	Average	SEM
3	766	306	13840	2697
5	796	101	16747	1254
7	550	108	16650	1989
7 (negative control)	6	1	13689	634

Supplementary Table 2. Total amount of neurons identified in the whole brain.

Entries show 5 samples for each condition.

Sample id	Condition	All neurons	1-tuned	2-tuned	3-tuned	4-tuned	5-tuned
hz04	3dpf	18104	1043	54	2	2	6
hz05	3dpf	14981	2181	51	9	6	4
hz12	3dpf	2346	362	1	0	0	0
hz13	3dpf	14467	834	10	2	2	5
hz14	3dpf	19303	1731	135	5	0	2
hz06	5dpf	20603	925	120	5	3	5
hz08	5dpf	19598	478	40	0	0	2
hz15	5dpf	15221	877	74	102	4	1
hz28	5dpf	13613	539	19	27	6	5
hz29	5dpf	14701	669	21	50	5	3
hz01	7dpf	23980	599	8	57	4	6
hz02	7dpf	16359	436	15	16	9	8
hz09	7dpf	14874	564	18	14	1	2
hz11	7dpf	17769	775	63	20	1	3
hz17	7dpf	10269	90	26	11	2	4
hz18	no_stim	16061	0	1	1	1	1
hz19	no_stim	13386	1	1	1	0	2
hz20	no_stim	13247	1	1	2	3	2
hz21	no_stim	11689	0	0	1	2	0
hz22	no_stim	14062	1	0	2	0	7
hz33	etoh_7dpf	15282	80	6	4	2	0
hz34	etoh_7dpf	16740	368	132	24	1	3
hz35	etoh_7dpf	19178	336	38	18	9	0
hz36	etoh_7dpf	13645	265	22	4	4	5
hz37	etoh_7dpf	15861	163	51	10	3	0

Supplementary Table 3. Total amount of neurons identified in the forebrain.

Entries show 5 samples for each condition.

Sample id	Condition	Forebrain	1-tuned	2-tuned	3-tuned	4-tuned	5-tuned
hz04	3dpf	3468	303	7	2	2	0
hz05	3dpf	2363	776	5	2	2	2
hz12	3dpf	220	58	1	0	0	0
hz13	3dpf	3671	179	2	0	2	3
hz14	3dpf	3643	714	32	1	0	1
hz06	5dpf	7095	596	4	0	1	1
hz08	5dpf	6546	225	0	0	0	2
hz15	5dpf	3495	355	14	2	0	0
hz28	5dpf	4925	304	6	5	2	4
hz29	5dpf	4871	257	3	0	1	3
hz01	7dpf	8046	363	4	2	2	3
hz02	7dpf	4160	82	3	0	3	4
hz09	7dpf	6296	266	1	1	1	0
hz11	7dpf	5292	293	2	2	0	0
hz17	7dpf	3788	54	0	0	0	3
hz18	no_stim	6949	0	1	1	1	1
hz19	no_stim	4493	1	1	1	0	2
hz20	no_stim	4602	1	1	2	3	2
hz21	no_stim	4260	0	0	1	2	0
hz22	no_stim	4448	1	0	2	0	7
hz33	etoh_7dpf	3997	11	0	2	0	0
hz34	etoh_7dpf	3354	62	1	1	1	0
hz35	etoh_7dpf	2816	21	11	12	2	0
hz36	etoh_7dpf	2528	6	0	0	1	2
hz37	etoh_7dpf	3580	11	4	0	1	0

Supplementary Table 4. Total amount of neurons identified in the midbrain.

Entries show 5 samples for each condition.

Sample id	Condition	Midbrain	1-tuned	2-tuned	3-tuned	4-tuned	5-tuned
hz04	3dpf	3665	660	29	0	0	0
hz05	3dpf	3554	1163	41	7	2	0
hz12	3dpf	741	288	0	0	0	0
hz13	3dpf	4378	632	7	0	0	2
hz14	3dpf	4392	927	94	1	0	1
hz06	5dpf	6323	298	116	3	0	2
hz08	5dpf	3502	203	40	0	0	0
hz15	5dpf	5847	513	59	100	4	0
hz28	5dpf	2603	171	12	20	2	0
hz29	5dpf	3889	402	17	48	2	0
hz01	7dpf	5936	166	2	52	2	3
hz02	7dpf	3263	218	1	3	5	4
hz09	7dpf	2468	242	13	8	0	2
hz11	7dpf	4653	418	46	7	1	3
hz17	7dpf	1614	29	21	11	0	1
hz18	no_stim	1817	0	0	0	0	0
hz19	no_stim	1972	0	0	0	0	0
hz20	no_stim	1103	0	0	0	0	0
hz21	no_stim	2620	0	0	0	0	0
hz22	no_stim	1813	0	0	0	0	0
hz33	etoh_7dpf	5306	68	4	1	1	0
hz34	etoh_7dpf	4442	292	123	22	0	0
hz35	etoh_7dpf	6022	274	26	3	3	0
hz36	etoh_7dpf	4951	242	22	0	1	1
hz37	etoh_7dpf	4325	131	39	9	0	0

Supplementary Table 5. Total amount of neurons identified in the hindbrain.

Entries show 5 samples for each condition.

Sample id	Condition	Hindbrain	1-tuned	2-tuned	3-tuned	4-tuned	5-tuned
hz04	3dpf	10971	660	29	0	0	0
hz05	3dpf	9064	1163	41	7	2	0
hz12	3dpf	1385	288	0	0	0	0
hz13	3dpf	6418	632	7	0	0	2
hz14	3dpf	11268	927	94	1	0	1
hz06	5dpf	7185	298	116	3	0	2
hz08	5dpf	9550	203	40	0	0	0
hz15	5dpf	5879	513	59	100	4	0
hz28	5dpf	6085	171	12	20	2	0
hz29	5dpf	5941	402	17	48	2	0
hz01	7dpf	9998	166	2	52	2	3
hz02	7dpf	8936	218	1	3	5	4
hz09	7dpf	6110	242	13	8	0	2
hz11	7dpf	7824	418	46	7	1	3
hz17	7dpf	4867	29	21	11	0	1
hz18	no_stim	7295	0	0	0	0	0
hz19	no_stim	6921	0	0	0	0	0
hz20	no_stim	7542	0	0	0	0	0
hz21	no_stim	4809	0	0	0	0	0
hz22	no_stim	7801	0	0	0	0	0
hz33	etoh_7dpf	5979	68	4	1	1	0
hz34	etoh_7dpf	8944	292	123	22	0	0
hz35	etoh_7dpf	10340	274	26	3	3	0
hz36	etoh_7dpf	6166	242	22	0	1	1
hz37	etoh_7dpf	7956	131	39	9	0	0

Supplementary Table 6. Comparison of age-related change in number-selective neurons in subregions of the forebrain.

Kruskal–Wallis test across age groups (n=5) for each region, adjusted for multiple comparisons using Bonferroni correction with alpha = 0.00625.

Region	F value	p value
Dorsal Telencephalon (Pallium)	3.42	0.18
Eminentia Thalami	6.74	0.03
Hypothalamus	8.3	0.02
Posterior Tuberculum (Basal Part of Prethalamus and Thalamus)	0.82	0.66
Pretectum	0.26	0.88
Prethalamus (Ventral Thalamus)	1.46	0.48
Thalamus (Dorsal Thalamus)	4.02	0.13
Ventral Telencephalon (Subpallium)	5.89	0.05

Supplementary Table 7. Summary of confusion matrix score.

This table presents the fraction of prediction instances using performance metrics such as precision, recall, and F1-score. Precision indicates the proportion of correctly predicted positive observations to the total predicted positives (i.e., the accuracy of positive predictions). Recall (also known as sensitivity) represents the proportion of correctly predicted positive observations to all observations in the actual class (i.e., the ability to find all relevant instances). The F1-score is the harmonic mean of precision and recall, providing a single metric that balances both concerns.

	precision				recall				f1-score			
	3 DPF	5 DPF	7 DPF	7DPF EtOH	3 DPF	5 DPF	7 DPF	7DPF EtOH	3 DPF	5 DPF	7 DPF	7DPF EtOH
num_1	0.66	0.7	0.78	0.76	0.56	0.66	0.58	0.72	0.6	0.68	0.66	0.74
num_2	0.41	0.58	0.53	0.58	0.42	0.6	0.53	0.53	0.42	0.59	0.53	0.55
num_3	0.37	0.48	0.52	0.47	0.32	0.44	0.53	0.44	0.35	0.46	0.53	0.45
num_4	0.42	0.5	0.49	0.47	0.28	0.3	0.43	0.41	0.34	0.37	0.46	0.44
num_5	0.45	0.48	0.49	0.35	0.31	0.47	0.54	0.35	0.37	0.48	0.51	0.3
prestim	0.5	0.61	0.74	0.48	0.94	0.92	0.94	0.77	0.65	0.73	0.83	0.59

Supplementary Table 8. Summary of GeNEsIS parameters.

GEnerator of Numerical ElementS Images Software (GeNEsIS) is a custom program written in Matlab to create stimuli with different numerosity and controlled or constrain physical stimuli characteristics.

Parameter	Pixels	cm
Convex hull	100	4.84
Inter-distance	9	1.98
Constant radius	1.2	0.05808
Total area	26	5.72
Total perimeter	31	6.82
Radius variability	0.2	0.044
Mean inter-distance	1.1	0.242
Mean radius	0.8	0.176
Arena radius	10	2.2
Arena dimension (pixel)	10	2.2
Pixel_X screen	1280	16
Pixel_Y screen	720	9
